# Chronic Hepatitis D Virus Infection and Its Treatment: A Narrative Review

**DOI:** 10.3390/microorganisms12112177

**Published:** 2024-10-29

**Authors:** Poonam Mathur, Arshi Khanam, Shyam Kottilil

**Affiliations:** Institute of Human Virology, University of Maryland School of Medicine, Baltimore, MD 21201, USA; akhanam@ihv.umaryland.edu (A.K.); skottilil@ihv.umaryland.edu (S.K.)

**Keywords:** hepatitis D virus, hepatitis, treatment

## Abstract

More than 12 million individuals worldwide are chronically infected with the hepatitis D virus (HDV). HDV infection is the most severe form of viral hepatitis since it requires hepatitis B virus co-infection and accelerates progression to cirrhosis and hepatocellular carcinoma. Therefore, treatment modalities to slow the progression of the disease are essential but not yet available. In addition, no antiviral treatment to date has been shown to reliably eradicate HDV. Pegylated interferon (PEG-IFN) is the only universally used treatment to suppress HDV RNA replication and improve liver inflammation and fibrosis. This treatment can be completed in 12–18 months, but cure rates remain low, and success does not reliably increase with the addition of a nucleos(t)ide analog. PEG-IFN therapy is also limited by poor tolerability and multiple adverse effects, including neutropenia, thrombocytopenia, and neuropsychiatric symptoms. Newer antiviral therapies in development target unique aspects of HDV viral replication and show promising results in combination with PEG-IFN for long-term HDV RNA suppression. These newer antiviral therapies include buleviritide (which blocks HDV entry), lonafarnib (which prevents HDV assembly), and REP-2139 (which prevents HDV export). In this manuscript, we discuss the characteristics of HDV infection and review the new antiviral therapies approved for treatment and those under investigation.

## 1. Introduction

The hepatitis D virus (HDV), first discovered in 1977, is estimated to chronically infect 12 million people worldwide [1,2]. HDV requires concomitant hepatitis B virus (HBV) infection to establish productive infection [3]. The estimated prevalence of HDV among those with hepatitis B surface antigen (HBsAg) positivity is 0.8–15% [1,4,5]. HDV causes the most severe form of viral hepatitis (since it requires HBV co-infection), significantly accelerates the progression of liver cirrhosis, and increases the risk of hepatic decompensation and hepatocellular carcinoma (HCC) compared to HBV mono-infection [1,4,6,7,8,9]. Furthermore, HDV is an independent risk factor for liver-related and all-cause mortality in patients with chronic HBV infection [10]. This review provides a summary of HDV infection and its treatment.

## 2. Epidemiology

The true global prevalence of HDV is unknown due to the low proportion of HBsAg+ individuals that are screened for HDV, variations in screening guidance, limited epidemiologic data from several countries, and lack of standardized confirmatory anti-HDV antibody and PCR tests and availability of these tests [4,11,12]. There is high HDV endemicity in central and west Africa, central and east Asia, the Amazon Basin, eastern Europe, and the western Pacific region [1,4,12,13]. More specific prevalence estimates among HBsAg carriers show that Mongolia has the highest rate of HDV RNA prevalence (61%) [14]. Low HDV endemicity areas include northern Europe, North America, and Japan, likely in part due to hepatitis B vaccination campaigns and low HBV endemicity [1,3,4,13].

Like HBV, HDV is a blood-borne pathogen, so transmission and risk of infection relate to contact with blood from infected individuals. Since HDV infection requires the presence of HBV, vaccination against HBV can also prevent HDV [15]. Patients with HBV infection should be screened at least once for HDV co-infection [1,16]. Screening is particularly indicated in patients with HBsAg+ and low/undetectable HBV DNA but persistently high ALT [16]. Additional populations that are at increased risk for HDV infection and warrant repeated screening include people who inject drugs, sex workers, men who have sex with men, and those with iatrogenic exposures [1,16]. HIV and hepatitis C virus (HCV) co-infection also increase the risk of HDV due to similar modes of transmission, so patients with the former infections should also be screened for HDV infection [1,17]. However, high HDV prevalence outside of these populations is also possible; in an anti-HDV test implementation program in Barcelona, 60% of HDV RNA-positive patients had no risk factors identified [18].

## 3. HDV Virology and Immune Response

**Figure 1:**
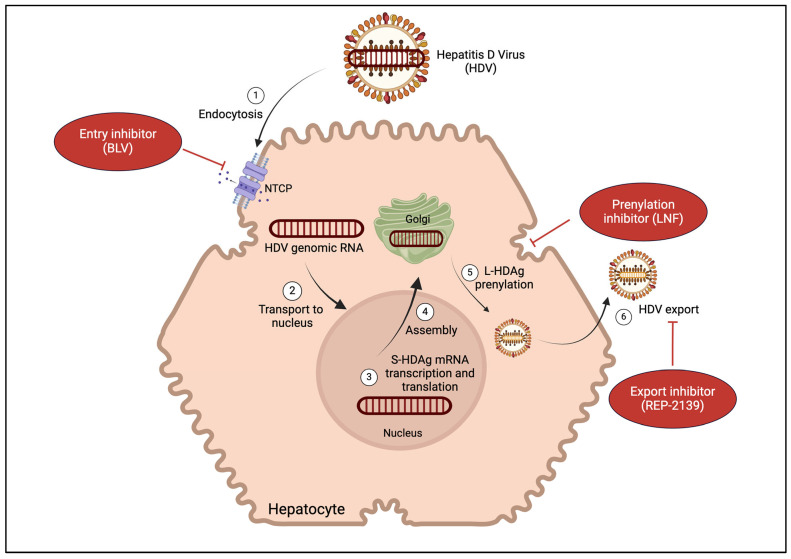
The hepatitis D virion and replication cycle. BLV = buleviritide. LNF = lonafarnib. NTCP = sodium/bile acid co-transporter. S-HDAg = small hepatitis delta antigen. L-HDAg = large hepatitis delta antigen. (Created with BioRender.com).

HDV is a single-stranded RNA virus consisting of a genome of 1672–1697 ribonucleotides [5,19]. Its genome is only slightly larger than that of a plant viroid, making it one of the smallest viruses known to infect humans [1,20]. HDV is a defective satellite virus that requires the presence of HBsAg for infection and viral replication; its envelope is derived from and composed of HBV surface proteins [3,5,19]. The HDV genome has only one actively transcribed open reading frame that codes for two forms of HDAg, large (L) and small (S). S-HDAg is necessary to initiate and maintain replication, while L-HDAg negatively regulates replication and triggers HDV to envelop the HBV surface proteins [5]. HDV uses HBsAg to enter hepatocytes via the sodium/bile acid co-transporter (NTCP) [21]; specifically, HDV binds to the pre-S1 domain of HBsAg so it can dock to NTCP. Once HDV enters and delivers its genome to the nucleus of the hepatocyte, S-HDAg-encoding mRNA is transcribed and translated [5]. After, the newly synthesized HDV RNA is transcribed to HDAg mRNA and uses a host enzyme, ADAR-1, to edit the HDAg mRNA, leading to the formation of L-HDAg [5]. L-HDAg is then prenylated by the enzyme farnesyltransferase [5]. This post-translational modification of L-HDAg—prenylation—is required for L-HDAg to interact with HBV envelope proteins and virion assembly [19]. This interaction can be blocked by prenylation inhibitors, which selectively target the assembly of the HDV virion [22]. Since HDV has limited protein-coding capacity, it deceives host machinery and is replicated by cellular RNA polymerases that are driven to replicate the HDV genome as though it were endogenous DNA, which makes finding therapeutic targets difficult (Figure 1) [23,24,25].

After infection, interferon (IFN) is the main innate immune mechanism to contain viral replication [5]. Unlike HBV, HDV induces an IFN response, which serves as a bridge to the development of adaptive immunity characterized by anti-HDV antibodies and HDV-specific T-cells [5]. In patients with active hepatitis, anti-HDV IgM antibody titers are high but lack neutralizing activity and do little to control viral replication [5]. The role of virus-specific T-cells is not well-defined for HDV infection, but they are presumed to be the drivers of viral control, similar to HBV and HCV infection [5].

There are eight distinct HDV genotypes that have different replication efficacies [5]. Genotype (GT) 1 is most common, whereas GT 2–8 are found in distinct geographic regions [3,5]. GT testing is not routinely undertaken, but there is emerging evidence that genotype may affect disease severity and treatment response [3,26,27].

## 4. Clinical Presentation and Natural History of Chronic HDV Infection

HDV can either be acquired as a co-infection with HBV or super-infection in already-established HBV infection [3]. Co-infection almost always results in spontaneous clearance of HDV without progression of the disease, similar to that seen in patients with HBV infection in the “immune control” phase [3]. Upon serologic evaluation, these patients would have a positive HDV antibody (Ab) but a negative HDV RNA. Super-infection, however, results in disease chronicity and rapidly progressive disease 90% of the time [3]. It is not well-known what causes the acceleration of liver disease, but it may be secondary to the direct cytopathic effects of the virus, especially S-HDAg, as well as the aberrant inflammatory cytokine release due to innate and adaptive immune responses. In multiple cohorts globally, it has been demonstrated that HDV replication is the major characteristic affecting disease severity. Chronic HDV infection from super-infection can suppress HBV DNA replication so that HDV is the dominant virus [3,12,24,25]. However, there are some cases where HBV replication predominates (30% of patients) or HBV replication and HDV replication are equal (15% of patients) [3].

People with HBV/HDV infection usually have chronic hepatitis, and persistent HDV viremia often accelerates liver fibrosis and increases the risk for cirrhosis, liver decompensation, and HCC [1,4,6,8,9,28]. Chronic HDV infection also increases the incidence of liver-related morbidity, need for liver transplantation, and liver-related mortality [3]. People with chronic infection may also present with persistent ALT elevation despite suppression of HBV DNA with nucleos(t)ide analog (NA) therapy [29]. Progression to cirrhosis can occur as quickly as within 5 years of infection and to HCC within 10 years [3]. Moreover, the risk of HCC is about three-fold higher in patients with HBV/HDV infection compared to those with HBV mono-infection [12]. Populations with increased risk of liver disease progression are listed in Table 1.

Patients with HBV/HDV infection who undergo liver transplantation are younger than those with HBV mono-infection and more likely to have decompensated liver disease. Fortunately, outcomes post-transplantation are similar between the two groups. In addition to clinical endpoints, patient-reported outcomes indicate that those with chronic HDV infection fare worse in their emotional and physical well-being compared to those with HBV infection alone.

Patients with HBV infection are monitored every 6–12 months for advancing fibrosis or HCC, and this is of even more importance for those with chronic HDV infection [28]. Monitoring is routinely carried out by ultrasonography, even if aminotransferase levels are normal [28]. An abnormal ultrasound of the liver should be followed up by computed tomography or magnetic resonance imaging of the liver [28]. Liver transplantation may be required due to the rapid progression of liver disease and/or decompensated cirrhosis [3]. Based on the U.S. national transplant database, patients with HBV/HDV infection receive liver transplants at younger ages than those with HBV only [3]. For example, in a U.S.-based retrospective study, patients with HDV undergoing liver transplants had a mean age of 52, and their HBV mono-infected counterparts had a mean age of 55.

## 5. Diagnosis and Liver Fibrosis Assessment

Diagnosis of HDV infection is made by screening for anti-HDV antibodies and using quantitative HDV RNA for confirmation (since anti-HDV IgM and IgG may remain positive after spontaneous clearance) [16,23,37,38]. Challenges to a uniform testing/diagnostic strategy are lack of provider knowledge regarding HDV, limited treatment options, and difficulty in accessing HDV-specific laboratory tests [3,4,12]. In addition, since standardized and validated real-time HDV RNA PCR assays are neither available worldwide nor FDA-approved, reflex testing, which would test for HDV IgM and IgG in all HBV-positive individuals, is not routine [23]. Additional items for consideration when evaluating a patient with HDV infection are shown in Table 2.

Liver fibrosis assessment is best carried out using transient elastography (TE) or liver biopsy. TE results in the context of HDV infection used to be difficult to interpret since there are no clear cut-offs for fibrosis; however, a recent study that compared TE to liver biopsy proposed numerical cut-offs for non-advanced liver fibrosis and cirrhosis, which may be of utility [39]. Non-invasive, serologic liver fibrosis tests used for HBV infection, such as the APRI and FIB-4 tests, do not perform well for HDV [40]. However, two non-invasive tests have been developed and validated for HDV: the delta fibrosis score and delta-4 fibrosis score [40,41]. Each score incorporates different variables to calculate the score and can be used as adjuncts to corroborate liver fibrosis staging derived from TE.

## 6. Treatment

Antiviral treatment is critical for reducing the morbidity and mortality associated with persistent HDV viremia [3,6,10]. Persistent suppression of HDV RNA is associated with improved clinical outcomes, including decreased liver-related complications [12,30]. The endpoint for successful treatment response is disputed, but most studies use undetectable or ≥2 log drop in HDV RNA 24 weeks after stopping treatment [16,42,43]. Treatment should normalize ALT levels, indicating a resolution of liver inflammation [16]. Loss of HBsAg is critical for the resolution of HDV infection and improves survival; therefore, tracking HBsAg levels may be a useful guide for when to stop treatment [44,45,46,47,48,49]. Accordingly, low HBsAg levels at baseline are a predictor of treatment response [44]. Unfortunately, most patients with chronic HBV infection do not lose HBsAg, making HDV RNA suppression difficult for co-infected patients [28]. A summary of approved and investigational treatments is found in Table 3.

### 6.1. Pegylated Interferons

The first treatment for HDV infection was pegylated-interferon-alpha (PEG-IFNa) for 48 weeks. Interferon is thought to interfere with HDV replication by inhibiting cell-to-cell spread during cell division and reducing the risk of liver decompensation, even if there is no virologic response. PEG-IFNa is a type I interferon administered subcutaneously (SC) (usually weekly) [6,50]. It has a sustained viral response rate (SVR) of 23–57% when treatment is administered for 48 weeks [16]. ALT normalization usually follows a virologic response, as well as improvement in liver histology [16,62]. Some patients lose HBsAg with 12–18 months’ treatment of PEG-IFNa, but sustained HBsAg loss usually requires prolonged treatment [49,62]. Studies of ≥2 years of treatment with PEG-IFNa have found higher rates of sustained HDV RNA (58%) and HBsAg clearance (33%), and improvements in liver histology [45,63]. Relapse of HDV viremia has been seen in about 50% of patients even after prolonged PEG-IFNa treatment, occurring up to 9 years after the completion of therapy [47,50,63,64]. Thus, the optimal treatment duration of PEG-IFNa is yet to be established [62,65]. Unfortunately, PEG-IFNa is contraindicated in advanced liver disease or major extrahepatic comorbidities [23]. It is also associated with neuropsychiatric and hematologic side effects, as well as paradoxical increases in aminotransferases, requiring frequent monitoring [23,45,50].

Success with PEG-IFNa is not enhanced by the addition of HBV-targeting NAs, as seen in randomized, placebo-controlled trials using adefovir or tenofovir disoproxil fumarate (TDF) in addition to PEG-IFNa or PEG-IFNa-2a [45,50,55,66]. In addition to the lack of efficacy enhancement, Anastasiou et al. noted that with the addition of an NA there were paradoxical increases in HBV DNA towards the end of treatment that were positively associated with HBsAg loss and HDV RNA suppression [66]. Moreover, biochemical markers of cell death (M30 and ALT) were higher during the HBV DNA peaks, suggesting that PEG-IFNa mediated death of hepatocytes may parallel increases in HBV DNA. In a systematic review and meta-analysis of randomized controlled trials using IFN and NAs, IFN and IFN+ NAs had high rates of viral relapse 24 weeks after the end of treatment, confirming that there is no apparent benefit of adding NAs to IFN [67]. Despite high rates of relapse, the AASLD and EASL recommend IFN+ NA treatment in HDV patients if they have cirrhosis or serum HBV DNA levels are elevated since there are significant benefits if, by chance, HDV suppression is achieved [16,23].

Due to the intolerable side effects associated with PEG-IFNa, a type III IFN, PEG-IFNl, was investigated in an open-label, proof-of-concept study, LIMT-1 [51]. Like IFNa, IFNl has a capacity for broad induction of antiviral activity with a similar downstream signaling pathway, but its receptors are mainly in gastrointestinal and respiratory tracts’ epithelial cells and not in hematopoietic cells. Due to the limited receptor distribution, the investigators of LIMT-1 assessed if the safety and tolerability of IFNl were better than IFNa. Thirty-three patients received IFNl 180 mg or 120 mg SC weekly for 48 weeks. The IFN 180 mcg group demonstrated higher efficacy: HDV RNA negativity at 24-week follow-up was 5/14 (36%) for participants in the 180 mg and only 3/19 (16%) in the 120 mg group. There were no significant changes in HBsAg levels throughout the study for either treatment group, and there were no significant associations between the HBsAg levels, HDV RNA, or ALT. Common side effects were flu-like symptoms and elevated transaminases. Only one patient had a neuropsychiatric side effect. Though PEG-IFNl appeared more tolerable than PEG-IFNa in this trial, no further investigations are planned due to four patients experiencing hepatobiliary events that resulted in hepatic decompensation in a separate phase III trial [68].

### 6.2. Novel HDV-Specific Antivirals

HDV-specific antiviral therapies inhibit the HDV life cycle via three mechanisms [12]:

1. Blocking HDV particles from entering hepatocytes (buleviritide, BLV).
2. Preventing assembly of mature infectious HDV particles (lonafarnib, LNF).
3. Preventing export of HDV particles (REP-2139).

#### 6.2.1. Buleviritide (BLV)

BLV is a myristoylated, synthetic lipopeptide corresponding to the preS1 sequence of HBsAg. Essentially, BLV is a mimic of the functional lipopeptide pre-S1 of HBsAg that HDV uses to dock to the NTCP co-transporter. Therefore, by acting as a pre-S1 lipopeptide decoy, BLV blocks HDV from entering hepatocytes. Overall, BLV is well-tolerated without drug-related serious AEs or treatment discontinuations [52,53,55]. The most common AEs have been headache, pruritus, fatigue, eosinophilia, injection site reactions, upper abdominal pain, and arthralgia. Asymptomatic dose-dependent increases in bile acid levels also occur; bile acid level increases are expected since the NTCP functions as a bile acid transporter [53,54,55,56,69,70].

To assess efficacy, the first-in-human, randomized phase 1b/2a study of BLV included 24 patients 1:1:1 to receive BLV 2 mg, PEG-IFNa-2a, or a combination for 24 weeks. BLV was well-tolerated without serious adverse events (SAEs), and there were significant reductions in HDV RNA after 24 weeks, with a higher mean of on-therapy decline when BLV was combined with PEG-IFNa [52]. Viral kinetic modeling confirmed the strong, synergistic effect of BLV and PEG-IFNa-2a on both HBV and HDV RNA levels. Also, ALT normalized with BLV monotherapy in 6 of 8 patients. However, the primary endpoint, ≤0.5 log decrease in HBsAg, was not reached in any patient. 

MYR202 was a multicenter, parallel-group, randomized, open-label phase II study that assessed the efficacy of BLV at different doses for 24 weeks. Participants were randomized 1:1:1:1 for 2, 5, and 10 mg of BLV with TDF 245 mg once daily or TDF 245 mg once daily alone [53]. The study enrolled 120 patients, and 59 patients had cirrhosis. The primary endpoint was HDV RNA below the level of detection or ≥2 log IU/mL decline in HDV RNA at week 24. The results for participants with virologic responses were: BDV 2 mg + TDF, 15/28 (54%); BDV 5 mg + TDF, 16/32 (50%); BDV 10 mg + TDF, 23/30 (77%); and TDF 1/28 (4%) (*p* < 0.0001 for all BDV/TDF compared to TDF alone). Interestingly, efficacy was not dose-dependent. Liver biopsies from 22 participants at baseline and at the end of treatment were analyzed, and all showed decreased HDV RNA; however, 89% (49/55) of participants developed relapse after treatment discontinuation, with aminotransferase flares in 22% of patients, suggesting BLV efficacy with an NA is not sufficient for HDV suppression, unlike what was seen when BLV was combined with PEG-IFNa-2a. Similar to the BLV + PEG-IFNa-2a combination, there were no significant changes in serum HBsAg levels with BLV + NA.

A subsequent phase II study, MYR203, was a multicenter, open-label, randomized study that allocated 90 participants into six treatment groups: PEG-IFNa 180 μg weekly, BLV 2 mg daily + PEG-IFNa 180 μg weekly, BLV 5 mg daily + PEG-IFNa 180 μg weekly, BLV 10 mg + PEG-IFNa 180 μg weekly, and BLV 10 mg + TDF, or BLV 2 mg daily for 48 weeks [55]. Twenty-four weeks after the end of treatment, the highest rates of HDV RNA below the level of detection were in participants who were treated with BLV 2 mg daily + PEG-IFNa 180 μg weekly (53%). Also, there were more BLV 2 mg dose participants with ≥1 log HBsAg log decrease (40% patients) than in the other groups, including those treated with 5 mg and 10 mg of BLV, confirming a lack of dose-dependent efficacy for BLV, as was suggested by MYR202. At follow-up, HDV RNA clearance persisted in patients who had decreased HBsAg.

BLV was also investigated in a randomized, phase III study, MYR301 [54]. There were 150 participants randomly assigned 1:1:1 to receive BLV 2 mg or 10 mg daily for 144 weeks or no treatment for 48 weeks, followed by BLV 10 mg daily for 96 weeks (the control group). The primary endpoint, which was a combined response at week 48 of undetectable HDV RNA or level that decreased by ≤2 log IU/mL and normalization of ALT, occurred in 45% of participants in the BLV 2 mg group, 48% in the BLV 10 mg group, and 2% in the control group (*p* < 0.001 for comparison with control group). ALT normalized in 12% of participants in the control group, 51% in the BLV 2 mg group, and 56% in the BLV 10 mg group. As seen in phase II studies, there was no efficacy advantage of BLV 10 mg over 2 mg for HDV RNA suppression (20% vs. 12%, respectively, *p* = 0.41). HBsAg level loss or level decrease by ≥1 log IU/mL did not occur in either group at week 48. In the ongoing study of MYR301 to week 96, out of 150 participants who did not have a virologic response at week 24, 43% achieved a virologic response at week 96. Biochemical response also occurred, but this was often independent of virologic response [71]. Also, post-study analyses of liver biopsy samples from patients in the MYR202, MYR203, and MYR301 trials show that BLV effectively decreased the number of HDV-positive hepatocytes, which results in a concomitant decrease in transcriptional levels of inflammatory chemokines and interferon-stimulated genes, compared to placebo, reinforcing the notion that long-term use of BLV for more than 24 weeks may increase HDV SVR rates [72].

MYR204 was a phase 2b, open-label trial comparing the efficacy of (1) PEG-IFNa-2a (180 μg weekly) for 48 weeks, (2) BLV 10 mg for 96 weeks, and (3) BLV (2 mg or 10 mg daily) in combination with PEG-IFNa-2a (180 μg weekly) for 48 weeks, followed by the same dose of BLV alone for another 48 weeks [73]. The primary endpoint was HDV RNA levels 24 weeks after the end of treatment, and the primary comparison was between the BLV 10 mg alone and BLV 10 mg + PEG-IFNa-2a groups. The combination of BLV 10 mg + PEG-IFNa-2a was superior in regard to undetectable HDV RNA at week 24 (34 percentage point difference; 95% confidence interval 15 to 50; *p* < 0.001).

Last, BLV has been investigated in prospective studies of patients with HDV infection and compensated cirrhosis [56,74]. In a cohort of 18 Caucasian patients who received BLV 2 mg daily for 48 weeks, there were no participants who developed hepatic decompensation or HCC. The regimen was well-tolerated, with no discontinuations for AEs or symptoms of increased bile acids. Virologic response (HDV RNA below the level of detection or decline of ≥2 log IU/mL at week 48) was met in 78% (14/18) of participants. Most participants (15/18, 83%) also had ALT normalization. In another study, two patients with HDV-related compensated cirrhosis were treated with BLV 10 mg daily for up to 3 years. Virologic and biochemical responses were maintained, and in one patient, liver function tests improved, and esophageal varices disappeared. Though HBsAg levels declined, they did not disappear completely. These data suggest BLV can be used to safely treat HDV infection in patients with cirrhosis who have contraindications to IFN treatment.

Overall, BLV seems to be well-tolerated and without dose-dependent efficacy on HDV RNA decline. HBsAg levels are unaffected by BLV monotherapy, but the addition of PEG-IFNa seems to enhance initial HDV RNA decline and decrease HBsAg; this effect may be due to PEG-IFN enhancing the kinetics of viral decline [75]. A systematic review and meta-analysis of randomized controlled trials confirms IFN + BLV’s superiority to other HDV treatment regimens (including BLV alone) in HDV suppression at least 24 weeks after the end of treatment [67]. In addition, BLV seems to have a high barrier to virologic resistance, based on post-study analysis of non-responders and those with virologic breakthroughs after BLV monotherapy in the MYR202 and MYR 301 studies [76].

As monotherapy, the 2 mg dose of BLV has been approved for HDV treatment in Europe as of July 2023 [23]. In the United States, the FDA granted BLV a breakthrough therapy and orphan drug designation in October 2022, but it has not been fully FDA-approved [77]. After European approval, several real-world studies support BLV’s safety and efficacy. A large, multicenter, real-world cohort of 114 patients in Germany treated with BLV 2 mg (without IFN) confirmed the efficacy of the drug, with virologic response in 76% (87/114) of cases and declines in ALT levels [78]. Other real-world studies show BLV’s safety and efficacy in treating HDV in patients with HIV co-infection and advanced liver disease, with normalization of ALT between 2 and 6 months of treatment [79,80,81]. In addition, economic models suggest that BLV can prevent significantly more liver events than PEG-IFNa in patients with chronic HDV infection and contribute to cost savings through reductions in liver complications [82]. BLV is a promising HDV treatment, but a remaining challenge with BLV treatment is the lack of a decline in HBsAg and identifying predictors of non-response to treatment; it has been suggested that low body mass index and high alpha-fetoprotein prior to treatment are possible predictors of delayed treatment response [81].

#### 6.2.2. Lonafarnib (LNF)

LNF inhibits farnesyl transferase, the enzyme necessary for the prenylation modification of L-HDAg, thus preventing the assembly of HDV virions. LNF is an oral capsule first investigated in a proof-of-concept, double-blind, placebo-controlled study for antiviral efficacy against HDV [57]. The study randomized 14 participants to receive LNF 100 mg twice daily (BID) or LNF 200 mg BID for 28 days with 6 months’ follow-up. The primary efficacy endpoint was a decrease in HDV RNA. There was a mean log HDV RNA decline of −0.73 log IU/mL in the LNF 100 mg group and a decline of −1.54 log IU/mL in the LNF 200 mg group. LNF serum concentrations correlated with HDV RNA changes, but there were no changes in serum HBsAg levels. AEs associated with LNF were mainly mild, consisting of nausea, diarrhea, abdominal bloating, and weight loss, which were more profound in the 200 mg group.

LOWR HDV-1 was a single-center, phase II proof-of-concept study exploring optimal LNF dosing regimens [58]. Fifteen participants received LNF 200 mg or 300 mg BID for 12 weeks, LNF 100 mg three times daily for 5 weeks, LNF 100 mg BID + PEG-IFNa-2a 180 mg weekly for 8 weeks, or LNF 100 mg BID + ritonavir (RTV) 100 mg daily for 8 weeks (RTV is an oral protease inhibitor that can increase half-lives of other drugs due to CYP450 inhibition). The best antiviral responses were seen with the LNF+ PEG-IFNa combinations. LNF monotherapy caused gastrointestinal AEs, but RTV improved tolerability. The addition of RTV to LNF also resulted in substantial suppression of HDV RNA compared to the LNF monotherapy regimens. In two participants who received the 12-week regimens, there was a transient, post-treatment ALT elevation that was associated with suppression of HDV RNA and subsequent ALT normalization (like that seen with PEG-IFNa + NA). This was not seen in patients with LNF < 12 weeks and was not due to HBV reactivation. The LOWR HDV-1 study showed that (1) adding RTV to LNF gave better antiviral responses and tolerability, (2) PEG-IFNa-2a in combination with LNF was more effective than PEG-IFNa-2a monotherapy, and (3) transient ALT flaring may be beneficial and therapeutic.

The LOWR HDV-2 study expanded on the findings of the LOWR HDV-1 study to investigate varying doses and durations of LNF + RTV [59]. In this single-center, open-label, non-randomized, phase II study, 55 participants were enrolled into three treatment groups to receive high-dose LNF (LNF ≥ 75 mg BID + RTV for 12 weeks), all-oral low-dose LNF (LNF 25 or 50 mg BID + RTV for 24 weeks), or combination low-dose LNF with PEG-IFNa (LNF 25 or 50 mg BID + RTV + PEG-IFNa for 24 weeks). The primary endpoint was ≤2 log decline or HDV RNA below the lower limit of quantification at the end of treatment. The primary endpoint was reached in 46% (6/13) and 89% (8/9) of participants with the LNF 50 mg BID + RTV and LNF (25 or 50 mg BID) + RTV+ PEG-IFNa regimens, respectively. Similar to the LOWR HDV-1 study, multiple patients had post-treatment ALT increases, but these were well-tolerated and followed by HDV RNA suppression and ALT normalization. More gastrointestinal AEs were in the high-dose LNF group.

The LNF studies confirmed that the addition of PEG-IFNa enhanced antiviral efficacy. The LOWR HDV-2 study also found that adding RTV to PEG-IFNa had the most efficacy and tolerability for participants. A follow-up pharmacokinetic modeling study confirmed that LNF combined with RTV was the most efficient to achieve antiviral efficacy without increasing AEs [83]. Though LNF + RTV + PEG-IFNa is well-tolerated and effective, RTV’s potential to induce drug-drug interactions via CYP450 inhibition may present issues with concomitant medications. After completion of phase 3 trials with LNF, it was granted orphan drug designation in the U.S. and Europe. A remaining challenge in LNF’s efficacy is that it seems limited to suppression of HDV RNA with no to little changes in HBsAg levels.

#### 6.2.3. REP-2139

REP-2139, a nucleic acid polymer, prevents the export of HDV particles. REP-2139 was investigated in the open-label, non-randomized phase II trial called REP 301 [60]. This trial enrolled 12 patients with HBV/HDV co-infection in Moldova. Participants received 63 weeks of treatment with a 1-year follow-up. The treatment regimen was REP-2139 500 mg intravenous (IV) weekly for 15 weeks, REP-2139 250 mg IV + 250 mg PEG-IFNa-2a weekly for 15 weeks, then PEG-IFNa-2a 180 mg monotherapy weekly for 33 weeks. The most common AEs during REP-2139 monotherapy were pyrexia and chills. However, the safety and tolerability of the combination regimen were similar to PEG-IFNa-2a monotherapy. A majority (11/12, 92%) of participants had RNA suppression during treatment, 9 (75%) at the end of treatment, and 7 (58%) at follow-up. Long-term follow-up of REP 301 assessed safety, tolerability, and HDV functional cure at 3.5 years of follow-up [61]. No abnormal liver or kidney abnormalities were seen, HDV RNA suppression persisted in 7 of 11 participants, and HBsAg seroconversion was seen in 5 participants. More clinical investigations of this molecule are needed, but it shows promise if it can suppress HDV RNA and lead to HBsAg loss.

## 7. Conclusions and Future Directions

BLV, LNF, and REP-2139 studies support their safety and efficacy, especially when combined with PEG-IFNa, suggesting that magnifying the endogenous immune response is vital for enhancing viral kinetics and HDV RNA clearance. However, the need for PEG-IFNa is also a downside due to the adverse effects of this therapy. Moreover, studies with these antiviral agents have not elucidated a fully reliable virologic endpoint to therapy since they all used different endpoints, making it difficult to compare their efficacy [12]. To date, BLV is the only approved HDV-specific antiviral. A challenge remains in finding an antiviral therapy that suppresses HBsAg, since this seems crucial to persistent suppression of HDV RNA. For this reason, there may be room for development of other antivirals, including REP-2139.

In addition to the treatment challenges facing HDV, several challenges remain to limit HDV infections in the United States (U.S.) and worldwide (Table 4). First, there is a paucity of data on HDV’s prevalence or genotype distribution, and there are no established cohorts of patients. Second, clinicians do not routinely screen for HDV since there is a lack of knowledge regarding who should be screened and which diagnostics are available. In addition, some clinicians do not screen for HDV since no optimal HDV treatment is available. Third, the currently universally-used treatment—PEG-IFNa—has limited efficacy. Last, if an antiviral drug is approved, it is unclear if patients would be amenable to treatments due to drug-drug interactions, the need for daily injections, long durations of treatment with adverse events, and low potential for cure. Ultimately, these concerns will need to be addressed to understand the scope of the impact that these developing therapies have on curing and reducing the significant morbidity and mortality associated with HDV infection.

## Figures and Tables

**Table 1:** Patient groups infected with hepatitis D virus (HDV) at higher risk for liver disease progression [6,9,10,23,30,31,32,33,34,35,36]. HCV = hepatitis C virus. HBV = hepatitis B virus. NA = nucleos(t)ide analog.

Patient Group
Chronic HDV infection with persistent viremia
Older age
Male sex
HIV or HCV co-infection
High serum HBV DNA levels
Elevated gamma-glutamyl- or aminotransferase levels despite NA treatment
Chronic liver disease and cirrhosis
Cofactors of chronic liver injury, including alcohol abuse, obesity, and diabetes

**Table 2:** Evaluation of a patient with chronic hepatitis D virus (HDV) infection. Since no clear guidelines for surveillance of patients with HDV infection are available, clinicians should follow guidelines for HBV infection [28]. ^a^ No clear cut-offs for transient elastography (TE) have been established due to the paucity of data regarding TE use in HDV infection.

Laboratory Tests
Anti-HDV antibodies Quantitative HDV RNA Screening for HIV, hepatitis C virus, hepatitis A virus immunity Complete blood count Complete metabolic panel (glucose, electrolytes, and assessment of renal and liver function, including measurement of liver enzyme levels)
**Liver Fibrosis Evaluation**
Liver biopsy Transient elastography (TE) ^a^
**Health Maintenance**
Hepatocellular carcinoma screening with ultrasonography, computed tomography, or magnetic resonance imaging every 6–12 months Hepatitis A vaccine

**Table 3:** Mechanism of action and adverse effects of major therapies available for chronic hepatitis D virus (HDV) infection. NTCP = sodium/bile acid co-transporter. HDAg = large hepatitis D antigen. BID = twice daily. RTV = ritonavir. HBsAg = hepatitis B surface antigen. HBV = hepatitis B virus.

Drug	Mode of Delivery and Dose	Mechanism of Action	Considerations for Use	Adverse Effects	Treatment Considerations
Pegylated-IFN and pegylated-IFNa-2a	Subcutaneous injection: 180 mg/week for 12–18 months	Binds to type I interferon receptors, activating immunomodulatory and antiviral proteins.	Most used for HDV treatment	Increase in transaminases, abnormal hematologic laboratory results, fatigue, flu-like symptoms, and psychiatric symptoms [45,50].	Sustained virologic response ranges from 23 to 57% and does not increase with the addition of nucleoside analog [16].
Pegylated-IFNl	Subcutaneous injection: 180 mg or 120 mg/week for 48 weeks	Binds to type III interferon receptors, activating immunomodulatory and antiviral proteins.	Investigational	Flu-like symptoms, increase in transaminases, and psychiatric symptoms [51].	Investigated in proof-of-concept study only.
Buleviritide	Subcutaneous injection: 2 mg, 5 mg, or 10 mg	Blocks HDV docking to NTCP and hepatocyte entry.	Investigational, not yet FDA-approved.	Headache, pruritis, fatigue, eosinophilia, injection site reactions, upper abdominal pain, arthralgia, asymptomatic bile salt increase, and increase in transaminases [52,53,54].	Maximal efficacy when combined with PEG-IFNa. Reduces HDV RNA levels and normalizes ALT [52,55]. Efficacy not influenced by the presence of cirrhosis [56]. Daily injection may limit adherence.
Lonafarnib	Oral: 25 mg, 50 mg, 75 mg, 100 mg, 200 mg, or 300 mg BID	Inhibits L-HDAg prenylation.	Investigational, not yet FDA-approved.	Nausea, diarrhea, abdominal bloating, weight loss, and ALT flaring [57,58].	Maximal efficacy and tolerability when combined with RTV and PEG-IFNa [58,59]. RTV is a CYP450 substrate, causing drug-drug interactions.
REP-2139	Intravenous: 250 mg or 500 mg	Inhibits export/secretion of HDV from hepatocytes.	Investigational, not yet FDA-approved.	Pyrexia and chills [60].	Combined with PEG-IFNa for efficacy without compromising tolerability [60]. May remove integrated HBV DNA from the liver [61].

**Table 4:** Future directions for HDV research. HDV = hepatitis D virus.

Area of Interest	Research Questions about HDV
Epidemiology	How can global HDV prevalence estimates and associations be more accurately defined? Why do certain population groups report higher prevalences of HDV?
Genotypes	What is the relevance of genotypes on the natural history of HDV infection? Do genotypes affect treatment outcomes?
RNA measurement and fibrosis detection	How can the sensitivity and specificity of HDV RNA assays be improved? Can numerical cut-offs obtained from transient elastography stage liver fibrosis in HDV-infected individuals accurately?
Immunological parameters	What are the immunological mechanisms associated with disease progression and severity? What are the immune correlates of HDV clearance or functional cure? Are there immunological markers associated with disease severity and functional cure that can be monitored during HDV treatment?
Antiviral treatment	Can there be a common antiviral that can efficiently inhibit both HBV and HDV infection and effectively eliminate the chances of viral rebound? What endpoint should be taken into consideration to stop antiviral treatment without the risk of rebound viremia? What are the factors contributing to treatment non-response? Should therapies in development also target the host immune response to control viral replication?
